# Top-k Bottom All but *σ* Loss Strategy for Medical Image Segmentation

**DOI:** 10.3390/diagnostics15172189

**Published:** 2025-08-29

**Authors:** Corneliu Florea, Laura Florea, Constantin Vertan

**Affiliations:** Image Processing and Analysis Laboratory (LAPI), National University of Science and Technology Politehnica Bucharest, 313 Splaiul Independenţei, 060042 Bucharest, Romania; corneliu.florea@upb.ro (C.F.); constantin.vertan@upb.ro (C.V.)

**Keywords:** top-k loss, bottom all but σ, burned skin area, ultrasound fetal image, MRI scan, semantic segmentation

## Abstract

**Background/Objectives** In this study we approach the problem of medical image segmentation by introducing a new loss function envelope that is derived from the Top-k loss strategy. We exploit the fact that, for semantic segmentation, the training loss is computed at two levels, more specifically at pixel level and at image level. Quite often, the envisaged problem has particularities that include noisy annotation at pixel level and limited data, but with accurate annotations at image level. **Methods** To address the mentioned issues, the Top-k strategy at image level and respectively the “Bottom all but σ” strategy at pixel level are assumed. To deal with the discontinuities of the differentials faced in the automatic learning, a derivative smoothing procedure is introduced. **Results** The method is thoroughly and successfully tested (in conjunction with a variety of backbone models) for several medical image segmentation tasks performed onto a variety of image acquisition types and human body regions. We present the burned skin area segmentation in standard color images, the segmentation of fetal abdominal structures in ultrasound images and ventricles and myocardium segmentation in cardiac MRI images, in all cases yielding performance improvements. **Conclusions** The proposed novel mechanism enhances model training by selectively emphasizing certain loss values by the use of two complementary strategies. The major benefits of the approach are clear in challenging scenarios, where the segmentation problem is inherently difficult or where the quality of pixel-level annotations is degraded by noise or inconsistencies. The proposed approach performs equally well in both convolutional neural networks (CNNs) and vision transformer (ViT) architectures.

## 1. Introduction

In recent years, the task of image segmentation—specifically, assigning a class label to each individual pixel in an image—has gained considerable importance within the field of medical imaging [1,2,3,4]. This growing attention is largely driven by the critical need for accurate, automated delineation of anatomical structures and pathological regions in clinical workflows. The dominant trend in addressing this task has shifted towards deep learning-based approaches, leveraging the power of advanced neural architectures to improve performance. These approaches primarily take the form of convolutional neural networks (CNNs) [5,6] or, more recently, vision transformers (ViTs) [7,8].

While deep learning has indeed brought significant advancements in medical image segmentation, the results are not uniformly satisfactory across all applications and datasets. Several challenges remain, including issues related to generalization across domains, annotation quality, and robustness to visual variability. In response to these limitations, numerous enhancements have been proposed in literature, targeting different components of the learning pipeline.

Some of these efforts have focused on refining the network architecture itself to better capture complex anatomical features. A representative example is the AtlasNet framework [9], which introduced architectural innovations tailored for medical image analysis. Other research directions have concentrated on rethinking the learning paradigm, proposing alternative training strategies to overcome dataset-specific limitations. These include the application of unsupervised domain adaptation techniques [10], which aim to bridge the gap between labeled and unlabeled domains, as well as contrastive learning approaches [4], which improve feature discrimination by leveraging similarity relationships among data samples.

Another direction of improvement is the modification of the loss function [2]. In this case, the most popular approach is to average the individual losses (e.g., mean square loss, cross-entropy loss, dice loss) over all examples by implementing the principle of Empirical Risk Minimization (ERM) [11]. The preference for the ERM is twofold: an efficient optimization algorithm and a solid theoretical base, as highlighted by [12]. In the ERM view, the individual losses are aggregated within their average, a method known as the Average Loss. An alternative to the Average Loss is to select the largest (Top-k) individual losses. In this way, one forms a more stringent criterion than the simple average and pushes the optimization to focus on hard examples, as emphasized by [13]. Recently, variants of the Top-k loss have been explored in image classification [14,15,16].

In this paper, we introduce a novel framework, along with an envelope mechanism, designed to aggregate the general loss function in a way that is tailored to specific challenges in medical image segmentation. The proposed framework is adaptive and highly flexible, allowing it to integrate seamlessly with a wide variety of models and learning strategies. In the context of semantic segmentation, we typically deal with two types of labels: those at the image level (indicating which classes are present in the image) and those at the pixel level (specifying the exact location of class regions through pixel-wise annotations).

**Figure 1:**
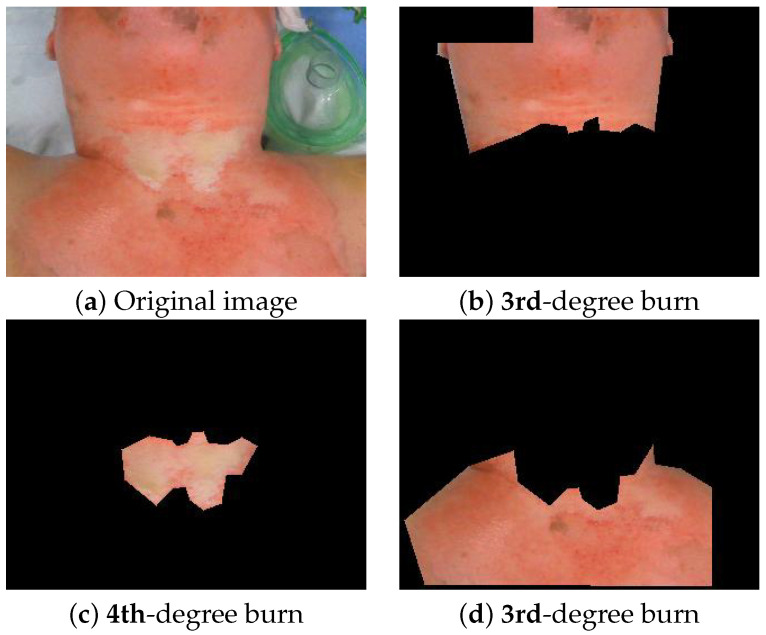
Example of burned skin area and marked regions from the BAMSI database. The full image (**a**) contains the entire scene (including background and normal skin areas) and the burned lesions has 3 sub-areas marked as being 3rd-degree burn (**b**,**d**), and 4th-degree burn (**c**). We name the degree of the burn as being an annotation at image level. The annotations have been done by an expert (plastic surgeon), but due to the limitations of the annotation tool, at the boundaries between regions, the individual pixel labels are problematic. Note the boundaries of the most serious burn (**c**).

To provide a more intuitive understanding of the motivation behind our approach, we begin by examining the specific case of burned skin area segmentation. This particular task is addressed using the BAMSI (Burned Area Multi Spectral Image) database, with representative examples shown in Figure 1. The annotations in this dataset were performed by an expert—specifically, a plastic surgeon with relevant domain knowledge. However, despite the expert involvement, the pixel-level annotations tend to be noisy, particularly near the boundaries of the annotated regions. This imprecision arises from several contributing factors. One is the intrinsic uncertainty and ambiguity associated with determining the exact transition between healthy and affected tissue, especially in images where contrast is poor or resolution is limited. Another source of imprecision comes from the annotation tools themselves, which often favor simplified shapes—such as straight lines or smooth curves—for the sake of annotation speed and efficiency. These tools may introduce bias or oversimplification near the borders of critical regions.

In contrast, the image-level labels in this task—indicating the severity or degree of a burn area—are considerably more reliable. These labels are usually derived from comprehensive clinical knowledge, with the expert often having direct access to the patient’s medical history and pathology reports, rather than relying solely on visual assessment of the image. As a result, image-level labels tend to carry higher semantic value and can be considered trustworthy.

From a broader perspective on segmentation tasks, it becomes evident that—depending on the nature of the problem—it may be beneficial to selectively ignore certain pixel-level information and instead rely more heavily on the image-level annotations. This is especially true in cases where the image quality or annotation precision is compromised. Conversely, hard or ambiguous examples at the image level, typically carry significant diagnostic value and are accurately labeled; as such, they should be given increased emphasis during training. The proposed framework is designed to make this distinction, adjusting the learning focus accordingly to improve performance across a range of medical segmentation scenarios.

Building on the observations discussed earlier, we propose a loss function strategy that leverages the ranking of individual loss values to guide learning. This approach is designed to be highly adaptable, allowing it to accommodate a range of segmentation scenarios in a fluid and principled manner. Our strategy draws inspiration from the Top-k loss formulation introduced by [15], which has previously demonstrated effectiveness in both binary and multi-class image classification tasks. In this work, we extend and adapt the classical Top-k loss to suit the specific requirements of semantic segmentation, particularly in the context of medical imaging.

It is important to highlight a key distinction between general image classification and medical image segmentation: while classification tasks, such as those involving datasets like ImageNet, often benefit from access to millions of labeled images, medical segmentation tasks typically operate with far fewer annotated samples—usually in the range of thousands. This limitation stems from the fact that medical annotation demands expert knowledge and careful, time-intensive manual labeling, often making the process significantly slower and more resource-intensive. Although the availability of annotated medical image data continues to grow, the pace remains considerably slower than in other image-based domains.

The core contribution of this paper is a novel and flexible loss function strategy for semantic segmentation in medical imaging, formulated within a parametric framework. The method is initially introduced and validated on the task of burned skin area segmentation using color images. However, to demonstrate the generalizability and robustness of the approach, we extend our experiments to include additional datasets spanning different imaging modalities, specifically MRI scans and ultrasound images.

A key feature of our method is its compatibility with a variety of deep learning architectures, including both convolutional neural networks (CNNs) and vision transformers (ViTs). To ensure stable training and to address potential convergence issues inherent in ranking-based selection mechanisms, we incorporate a smoothing procedure. This is implemented through differentiable approximations that allow the selection of top and bottom loss values to remain tractable and effective during gradient-based optimization.

The remainder of the paper is organized as follows: Section 2, we review related work and provide context for our contributions. Section 3 introduces the proposed formulation and outlines the loss function strategy in detail. Section 4 covers practical aspects of the implementation, including model training and dataset handling. Finally, the paper concludes with a comprehensive discussion of the experimental results, as well as a critical reflection on the strengths and limitations of the proposed method.

## 2. Previous Work

**Rank and top-k losses.** The recent interest in rank losses originates in prior attempts to explore the loss functions for binary classification, with an emphasis on structuring and smoothing the hinge loss in SVMs [17]. In parallel, studies like those by [13] and, respectively [18], have identified specific limitations of the average aggregate loss, particularly when dealing with imbalanced datasets. This has generated interest in exploring alternative aggregate loss formulations, like adopting the maximum individual loss as an overall measure, known as “Maximum Loss”. Building on this loss, ref. [15] developed the “ATk Loss” or Average Top-k Loss, which calculates the average from the largest top *k* individual losses. This type of loss proved to have superior performance over both “Average Loss” and “Maximum Loss” across various benchmarks. However, it faces issues with the derivatives in the learning stage. One approach to alleviate the problem with the derivatives was to smooth the overall loss using an approximation with polynomials [14]. Another proposed approach was the use of the so called Split Selection Network, where the raking is distributed over all layers [19]. All these proposals emphasize the role of data instances that produce the largest errors and they have been applied in image classification tasks.

Rank losses are the broader category which often form non-convex function. One popular example of a non-convex function, based on weighting, is the focal loss [20], which was proposed for the detection of clustered (i.e., hard) objects. The focal loss introduces a parametrization of the cross entropy to give higher weights, in a structured form, to all instances from a specific class, but still all examples within the dataset have to be used.

In the wider field of machine learning, there is a related area of research known as the data subset selection problem [21]. This focuses on selecting a subset from a large training dataset in order to train models, while aiming to minimize the average loss. Two contemporary learning strategies, namely curriculum learning [22] and self-paced learning [23], structure the training process in several iterative phases, moving from simple examples to more challenging ones based on individual loss levels. Thus, in these approaches, each training phase is characterized by the average aggregate loss of the chosen data subset.

The Top-k loss has been used in the comparative evaluation in the segmentation task, at the pixel level by [2]. They have found that a strategy that selects, by different means, the worst top-k pixels, can help in cases where annotations are very accurate at pixel level. In the same direction, ref. [24] ranked pixels values and, in the later iteration of learning, concentrated on the largest errors, i.e., the hardest pixels to classify. They approached problems with very accurate annotations at pixel level, which is not always the case.

Concluding, to our best knowledge, the rank loss in general and Top-k loss in particular has not been explored in the area of medical image segmentation. Also, strategies that can accommodate both using the top and, respectively, bottom values have not been proposed until now.

**Burned skin segmentation**. The main application of this paper is burned skin area segmentation. Burned area segmentation may be seen as a particular case of skin lesion image segmentation. From this perspective, ref. [25] used a superpixel based approach for the separation and classification of skin lesions. Yet, the wider field of skin lesions segmentation is better documented and has established benchmarks. The burned skin problem is less explored. Into this particular direction, ref. [26] used a convolutional network to separate and to automatically grade the burned skin from images. A more general overview of the usage of deep leaning for the assessment of burned skin by means of semantic segmentation has been offered by [27], but their presentation is directed more towards the medical auditorium, as they rather enumerate the available tools rather than discuss technical details. Ref. [28] went a step further, towards technicality, and matched the image with burned skin, segmented with a U–net, with the image of the healed area in order to predict the healing process. Overall, noting that the burned skin is a serious issue, even life-threatening with high morbidity and mortality [26], the accurate segmentation of the burned area is still needing contribution in both technical aspects and in consolidating frameworks. Furthermore, the task is difficult as the variability of the burned skin overlaps with the healthy skin and the background.

## 3. Method

In this section we will present the proposed methodology that focuses on a new aggregation of the loss function for semantic segmentation. The formal presentation of the method is complemented by shorter descriptions of the intuitive aspects.

### 3.1. Preliminaries, Principles

During the training of neural networks, the typical process begins with the selection of a specific type of individual loss. Examples of the loss may include cross-entropy loss, hinge loss, mean square loss, or logistic loss. This loss is computed for each data instance. Subsequently, these individual losses are aggregated over all data instances to compute their average according to the Empirical Risk Minimization principle [11]. This Average Loss function is applied extensively across a wide range of deep learning endeavors, including the semantic segmentation problems.

One particular characteristic of the ERM principle is the possibility to break its computation over subsets of data and to apply corrections over the model parameters given these partial accumulations, according to stochastic gradient principles. Nonetheless, recent studies [13,17] suggested that the ERM principle is not appropriate for all tasks; an example where ERM is sub-optimal is the Top-k error in image classification. To address some of the limitations, the “Maximum Loss” [13] was proposed and it was shown to offer interesting advantages.

The Maximum Loss [13] has the advantage of being a harder condition than the Average Loss, because it is always larger. If an optimization process drives the Maximum Loss towards zero, it also guarantees that the Average Loss is nullified. The disadvantage is related to its uniqueness (i.e., computed on a single data instance) and the fact that, without particular domain knowledge, there is no practical guarantee that the data instance which enables the maximal loss is not an outlier resulted from wrong data labeling. Building on the aforementioned advantages of the Maximum Loss, ref. [15] developed the “ATk Loss” or Average Top-k Loss, which calculates the average from the top *k* individual losses, demonstrating superior performance over both “Average Loss” and “Maximum Loss” across various benchmarks. However, the Average Top-k Loss function is not differentiable with respect to *k*.

In general, the maximal loss values are caused by outlying data instances. These can be either:

- Hard examples when the annotation process is accurate. In this case it is desirable to anchor the training on these outliers, especially in the later stages, forcing the model to predict them correctly.
- Noisy labels, when the annotation process is loose or inexact. In this case, the outliers should be avoided, since forcing the model to predict them correctly means pushing towards wrong directions and, thus, hurting the overall performance.

Concluding, prior information about the quality of annotation should be the driving force in how these outlier instances are used during the training process. Such prior information is typically part of the so-called domain knowledge. Here, we seek the formulation of an envelope of the individual loss functions that is able to accept specific domain knowledge and address both outlier data sources.

### 3.2. Formalization

#### 3.2.1. Notation

To ensure a clear understanding of the context and implications, we introduce several conventions of notation. In supervised learning, the training data, usually, comprises of an input set from the domain X and a target set from the domain *Y*. The training samples $x_{i}$ form a finite subset $X=x_{1},x_{2},\ldots ,x_{N}$ of the domain X and are associated with their $y_{i}$ labels; *N* is the cardinality of the data. Depending on the problem, the data instances differ: for image classification *N* is the number of images; for object detection *N* is the number of objects (i.e., bounding boxes). For segmentation, *N* is the total number of pixels within all images. The goal is to identify a predictor function f:X→Y within a function family *H*, with parameters w, that seeks to accurately forecast the target *y* for new inputs x; the actual prediction is $f_{w}\left(x\right)=\hat{y}$.

To measure the predictor’s performance, a loss function (which is considered to be of the error type, that is smaller is better, larger is worse.) $\ell :\hat{Y}\times Y\to R^{+}$ is introduced, where $\ell \left(f_{w}\left(x\right),y\right)$ measures the discrepancy between the predictor’s estimate $\hat{y}$ and the actual, desired value *y*. Training the predictor involves minimizing an objective function through gradient descent, which usually focuses on reducing the loss function.

For a set $L_{\left[\right]}=\ell_{\left[1\right]},\ell_{\left[2\right]},\ldots ,\ell_{\left[k\right]},\ldots ,\ell_{\left[N\right]}$, we use square brackets for indexes to mark that values are sorted in ascending order. $\ell_{\left[k\right]}$ is the *k*-th smallest value, while $\ell_{\left[N-k+1\right]}$ is the *k*-th largest value, satisfying $\ell_{\left[1\right]}\leq \ell_{\left[2\right]}\leq \ldots \ell_{\left[k\right]}\leq \ldots \ell_{\left[N-k+1\right]}\leq \ldots \leq \ell_{\left[N\right]}$.

#### 3.2.2. Non-ERM Approaches

The most popular way to compute the loss function is provided by the empirical risk minimization (ERM) principle [11], in which the statistical mean over the sample space is taken. Given that the (training) space is not available in its entirety, but only as a subset of samples, the discrete average is used:

(1) $$\ell_{avg}\left(f_{w},X\right)=\frac{1}{N}\sum_{i=1}^{N}\ell \left(f_{w}\left(x_{i}\right),y_{i}\right)=\frac{1}{N}\sum_{i=1}^{N}\ell_{\left[i\right]}$$

The learning in the training process is achieved by minimizing the $\ell_{avg}\left(f,X\right)$ loss and in the context of rank losses, the loss defined in Equation (1) is named the “Average Loss” and is the factual ERM implementation. An alternative is the “Maximum Loss” [13]:

(2) $$\ell_{max}\left(f_{w},X\right)=\max_{i\in \{1\ldots N\}}\ell \left(fw\left(x_{i}\right),y_{i}\right)=\ell_{\left[N\right]}$$

The maximum approach suffers from too small derivatives and issues convergence problems [14]. One leverage is to consider smoothing the loss function and one such proposal [15] is to use the average of the maximal top-k losses:

(3) $$\ell_{at_{k}}\left(f_{w},X,k\right)=\frac{1}{k}\sum_{i=N-k+1}^{N}\ell_{\left[i\right]}$$

Ref. [15] pointed that starting from Equation (3), if k=N, the average loss (empirical risk minimization) from Equation (1) is found, while if k=1 the maximum loss from Equation (2) is retrieved. In practical classification on ImageNet, ref. [15] showed that k=0.8N produces slightly better performance when measured by the Top-1 error and noticeable overall improvement for the Top-5 error.

If one wishes to select a particular group of *k* losses, from the ordered set $L_{\left[\right]}$, the overall loss may become:

(4) $$\ell_{select}\left(f_{w},X,k,i_{0}\right)=\frac{1}{k}\sum_{i=i_{0}}^{i_{0}+k-1}\ell_{\left[i\right]}$$

**Figure 2:**
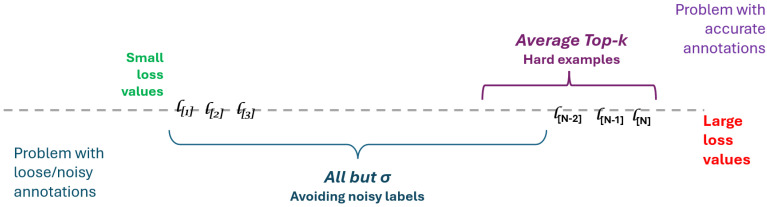
Impact of the annotation quality over the loss strategy. Loss values $\ell_{\left[i\right]}$ are in increasing order. We separate the case of accurate annotations (showed on the top of the drawing, over the dashed line) from the case of noisy annotations (showed below the dashed line). For accurate annotations, hard examples are selected by large loss values and need to be emphasized. For noisy annotations, large loss values mark noisy labels and the largest ones have to be ignored.

Here, by choosing $i_{0}=N-k+1$, the ”Average Top-k” from Equation (3) is retrieved. If $i_{0}=0$, the smallest *k* losses are used, retrieving the so-called Average Bottom-k ($AB_{k}$) [16]; yet, to our best knowledge, the later has only been formulated and not been used on a practical application. The formulation $\ell_{select}$ may be seen as a top-bottom simultaneous selection. We emphasize the following ideas, which are also illustrated in Figure 2:

- If the labels $y_{i}$ are accurate, then ”Average Top-k” is preferable because it focuses on hard examples.
- If the labels $y_{i}$ are noisy, then ”Average Bottom All but k” is preferable because it ignores *k* examples with potentially incorrect labels. This approach falls into the ”loss correction method”, using a re-weighting version, yet it is simpler and more elegant than previous approaches and for a review on the topic, we refer again the reader to the work of [29].

Let us define the indicator function I(a) as 1 when the proposition *a* is true, and 0 otherwise. Using it, the summation from Equation (4) may be rewritten such that it will select only those terms in the $\left[i_{0},\ldots ,\left(i_{0}+k\right)\right)$ range:

(5) $$\ell_{select}\left(f,X,k,i_{0}\right)=\frac{1}{k}\sum_{i=1}^{N}I\left(i_{0}\leq i\right)I\left(i<i_{0}+k\right)\ell_{\left[i\right]}$$

The selector could have been written as a single indicator function $I\left(i_{0}\leq i<i_{0}+k\right)=I\left(i_{0}\leq i\right)I\left(i<i_{0}+k\right)$, but writing it as a product and forming a boxcar function (also named “box” function), eases the explanation.

The selective loss expressed above is a simple difference of top-k losses (one of the top *k* values and one of the top $i_{0}+k$), and would be solved by optimization. Ref. [15] suggested a reformulation by re-writing it as the sum of shifted and weighted terms from the entire set. To determine the equivalent of *k* and $i_{0}$ they used an iterative gradient based algorithm. Yet, such a deployment for the envisaged segmentation problems failed to converge. As such, we identify the need of generating a continuous approximation of either the top-k or the selective loss, which is explained in the following subsection.

#### 3.2.3. Discontinuity and Approximation

The function from Equation (5) is discontinuous in $i_{0}$ and in $i_{0}+k$. These points create convergence problems in learning: changing significantly the selected data acts as a reset of the learning process and leads to oscillation.

To address this, we propose to approximate the indicator function by a member of the sigmoid function family $S_{\alpha }$, thus smoothing the discontinuities:

(6) $$I\left(i\geq k\right)=\left\{\begin{array}{cc} 1, & i\geq k\\ 0, & i<k \end{array}\right.\approx \frac{1}{1+e^{-\alpha (i-k)}}=S_{\alpha }\left(i,k\right)$$

where α is a parameter. This is a particular case of the generalized sigmoid. The larger the value of α, the more accurately $S_{\alpha }$ approximates the step-like indicator function. The sigmoid function is continuous, therefore it is smoothing the discontinuities.

We note that while many potential functions can be used. We preferred the formula from Equation (6) due to its popularity in machine learning.

The product indicator that forms a boxcar selector function in Equation (5) requires a two factor product:

$$\zeta_{\alpha }\left(i,i_{0},k\right)=S_{\alpha }\left(i,i_{0}\right)\cdot S_{\alpha }\left(-i,(i_{0}+k)\right)$$

(7) $$\ell_{select}\left(f,X,i_{0},k\right)\approx \frac{1}{N}\sum_{i=1}^{N}\zeta_{\alpha }\left(i,i_{0},k\right)\ell_{\left[i\right]}$$

**Figure 3:**
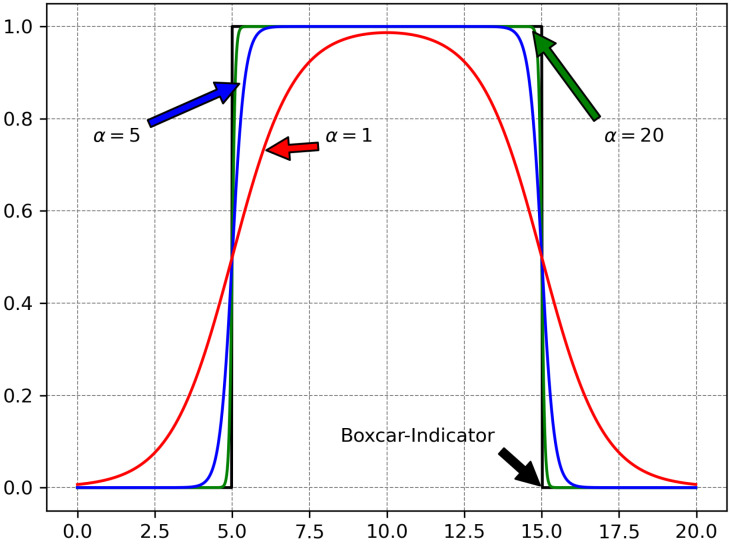
Approximation of the boxcar function with parametric sigmoids. We take as reference I(i≥5)I(i<15) (drawn with black) and plot $S_{\alpha }\left(i,5\right)S_{\alpha }\left(-i,-15\right)$ for α=1 with red, α=5 with blue, α=20 with green.

**Figure 4:**
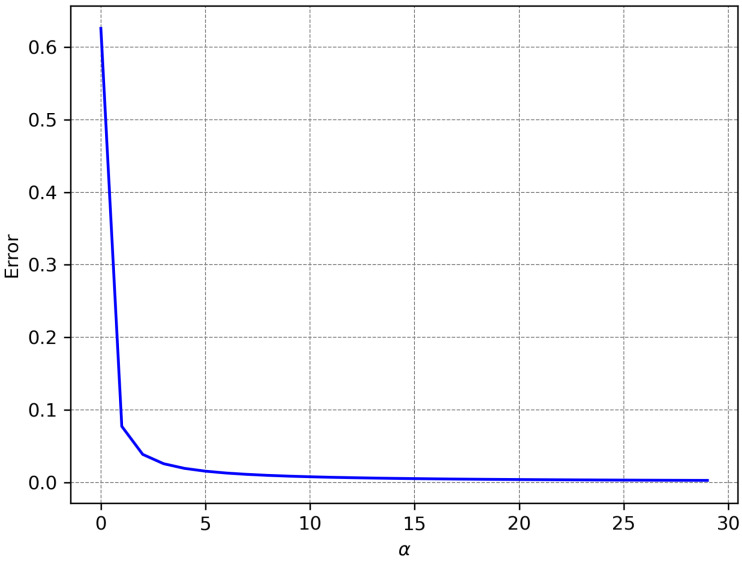
Maximum error of approximating the boxcar function represented in Figure 3 with the product of sigmoid-like functions for various α values.

Obviously $\zeta_{\alpha }\left(i,i_{0},k\right)\approx I\left(i_{0}\leq i<i_{0}+k\right)$. The quality of the approximation for various α values may be visualized in Figure 3, while the maximum error for the approximation of a boxcar selector may be seen in Figure 4.

#### 3.2.4. Proposed Loss Formulation for Segmentation

For the semantic segmentation, the number of instances *N* is given by the total number of pixels that have assigned segmentation labels. This assumes that each image in the database has the same dimensions (which is generally the case for medical imaging). Thus, the total number of pixels *N* may be decomposed into a product such that N=I×P: here *I* is the total number of images and *P* is the number of pixels in each image. Consequently, the summation of *N* terms from the ERM becomes a pair of nested sums. There, the outer sum ($\sum_{i=1}^{I}$) is over the *I* images (assumed to have the same resolution), while the inner sum ($\sum_{j=1}^{P}$) is over the *P* pixels within each image. Since in this particular case, $\ell_{\left[j\right]}$ is the loss associated to each single pixel, the Average loss (ERM) may be rewritten as:

(8) $$\ell_{avg}\left(f,X\right)=\frac{1}{I\cdot P}\sum_{i=1}^{I}\left(\sum_{j=1}^{P}\ell_{\left[j\right]}\right)$$

The loss strategy from Equation (5) becomes:

(9) $$\begin{array}{c} \ell_{select}\left(f,X,k_{i},i_{0},k_{p},j_{0}\right)=\\ \frac{1}{k_{i}\cdot k_{p}}\sum_{i=1}^{I}\left(\zeta_{\alpha_{I}}\left(i,i_{0},k_{i}\right)\sum_{j=1}^{P}\ell_{\left[j\right]}\zeta_{\alpha_{P}}\left(j,j_{0},k_{p}\right)\right) \end{array}$$

where $\zeta_{\alpha_{I}}\left(i,k_{i}\right)$ controls the selection at image level, while $\zeta_{\alpha_{P}}\left(j,k_{p}\right)$ controls the selection at pixel level within each image. In this case, one may also use the non-approximated form with I(·) instead of ζ(·) either at pixel level, or at image level. We will argue that from a computational point of view it makes sense to use the boxcar selector, I(·) at pixel level and its approximation by ζ(·) at image level.

#### 3.2.5. Combination with Various Losses

Until now, we have not detailed any particular choice for the individual losses. For the proposed approach, the individual loss function needs to provide continuous values at pixel level, such that the individual losses can be ranked.

The cross-entropy (CE) is a natural choice as it offers continuous values at pixel level, thus allowing the ranking of values and the selection of ranges.

On other hand, the popular segmentation loss, namely the Dice loss, provides a different challenge. In its standard form, the Dice Loss is computed over binary values (as the prediction map is transformed into a segmentation map) at pixel level, and thus it does not allow pixel loss ranking. An alternative that can take into account continuous values is offered by the continuous Dice Coefficient (cDC) [30], which for two regions expressed by sets (set *A* being binary and set *B* being continuous) is defined as:

(10) $$cDC=\frac{A\cap B}{\psi |A|+|B|},\;\psi =\frac{\sum_{i}a_{i}b_{i}}{\sum_{i}a_{i}sign\left(b_{i}\right)}.$$

In our case, the values from the two sets are the prediction $\hat{y_{j}}$ and the segmentation label annotations at every pixel $y_{j}$. These are considered in the probabilistic form ($\hat{y_{j}}$) and respectively one-hot encoding ($y_{j}$) at every pixel location *j*. The continuous Dice Coefficient becomes:

(11) $$cDC=\frac{1}{C}\sum_{c=1}^{C}\left(1-\sum_{j=1}^{P}\rho \left(\hat{y_{cj}},y_{cj}\right)\right),$$

where

$$\rho \left(\hat{y_{cj}},y_{cj}\right)=\frac{1}{\sum_{j=1}^{P}{\hat{y_{cj}}}^{2}+\sum_{j=1}^{P}y_{cj}^{2}}\hat{y_{cj}}y_{cj}$$

where $y_{cj}$ is the pixel label from location *j* corresponding to class *c* (out of *C*) and $\hat{y_{cj}}$ is the continuous prediction at the same location. Equation (11) contains $\rho (\hat{y_{cj}},y_{cj})$ values which are computed at every pixel and have continuous, thus rankable, values.

The overall loss for a pixel *j* becomes:

(12) $$\ell_{j}=\lambda_{CE}\ell_{CE}\left(\hat{y_{j}},y_{j}\right)+\lambda_{DC}(1-\rho \left(\hat{y_{j}},y_{j}\right)$$

where $\lambda_{CE}$ is the cross-entropy, while $\lambda_{CE}$ and $\lambda_{DC}$ are empirically determined constants.

### 3.3. Practical Aspects About“Bottom All but σ”

A key practical observation that deserves emphasis is the computational burden associated with ranking-based loss functions in prior work. In the context of image classification, such loss functions have typically been applied to datasets containing hundreds of thousands to millions of training examples. Sorting arrays of that size—even when using efficient algorithms with a computational complexity of O(NlogN)—can be computationally expensive and time-consuming.

In contrast, medical image segmentation datasets are significantly smaller in scale. Although there is a growing trend toward increasing the size and availability of medical imaging datasets for machine learning and computer vision applications, the rate of growth remains markedly slower than in general computer vision domains. This difference can be attributed to several factors.

First and foremost, the collection of a single medical imaging instance requires the occurrence of a real medical case, which may not be common in the general population. Even when such case arise, the primary concern is patient care and clinical decision-making, not data collection. Research use is, often, secondary. Secondly, high-quality annotation in medical imaging almost always requires expert input. Medical professionals, who are already burdened with clinical responsibilities, may have limited availability to contribute to the labor-intensive task of dataset annotation. This contrasts sharply with many general image datasets, where non-expert annotations are sufficient and more easily scalable.

Finally, it is essential to recognize the real-world implications of medical decision-making. Even when machine learning tools are used to assist with diagnosis or treatment planning, the final responsibility rests with the clinician. In contrast, errors in general image classification tasks—such as misidentifying an airplane in the CIFAR dataset—carry minimal real-world consequences. This clinical responsibility naturally constrains both data availability and annotation accuracy.

As a result, medical image segmentation datasets today are often one or more orders of magnitude smaller than those used in standard computer vision tasks. This reduced scale has an important practical implication: the computational overhead associated with sorting and ranking loss values is much lower, making ranking-based strategies more feasible in this domain.

Moreover, our proposed strategy does not rely on full sorting of the entire loss vector. Instead, after each training batch, the newly computed loss values are incrementally inserted into a pre-sorted structure. Assuming a batch size of *k*, this results in *k* insertions into an array of size N−k, yielding an overall complexity of O(k×N). This is generally much smaller than the cost associated with the backward pass during gradient computation. In some cases, if the loss value is sufficiently low, the corresponding gradients (e.g., from sigmoid layers) can be ignored altogether, and the backward update for those elements can be skipped. This selective update mechanism further reduces the computational overhead. Therefore, even if applied to larger medical imaging datasets than those used in our experiments, the proposed approach maintains efficiency and can offer computational advantages over standard empirical risk minimization (ERM) strategies.

Previous works on Top-k loss [14,15] have introduced optimization algorithms—such as block coordinate descent—to tune the hyperparameter $i_{0}$, which defines how many of the top-k losses to include in the objective. However, these methods were developed in the context of large datasets and do not specifically focus on emphasizing difficult examples. In contrast, when working with medical image segmentation tasks, selecting only a small subset of hard examples (i.e., using a small *k*) can hinder convergence and lead to unstable training.

To overcome this, we take a different approach at the pixel level. Since a single image can contain tens of thousands of pixels, full sorting is not only unnecessary but inefficient. Moreover, the vast majority of pixel-level predictions are valid and should be included in training. Thus, we adopt a dispersion-based strategy, denoted by the parameter σ, which determines a soft threshold for loss value inclusion. Instead of selecting a fixed number of pixels, we compute a statistical threshold: only those pixels whose loss values fall below $E[\ell_{i}]+\sigma$, where E[·] denotes the mean loss, are included. In this context, σ denotes the standard deviation of the pixel-level losses within an image. It is used to separate the low-loss pixels into two groups: those retained in the “Bottom” set and those excluded from training, thereby reducing the influence of overly easy or potentially noisy samples. In essence, this means we are using “all but σ” pixels.

Importantly, standard deep learning frameworks, such as PyTorch 2.7.0, allow for the computation of individual pixel-level losses before any aggregation at the image level. This enables straightforward implementation of our statistically-based filtering mechanism. In summary, our approach effectively balances precision and efficiency by avoiding unnecessary computation while still focusing the learning process on informative examples.

### 3.4. Convergence Aspects

Prior works on Top-k loss, such as [13,15], have approached convergence analysis by building upon the foundational work of [31]. In that foundational study, the theoretical framework assumes that the individual loss functions $\ell_{\left[i\right]}$ are either strictly linear or can be effectively approximated using piecewise linear functions. While elegant from a theoretical standpoint, this assumption is quite restrictive and rarely holds in practical applications. In many real-world scenarios—particularly in deep learning—the loss functions are inherently non-linear, and their behavior deviates significantly from any piecewise linear approximation.

Moreover, when the aggregation of losses involves more than just a handful of terms—especially when some of these terms violate the linearity assumption—the resulting behavior no longer conforms to the theoretical guarantees outlined in the original work. In such cases, the summed loss function may diverge from the expected behavior, particularly in small-sample regimes, which are common in medical image segmentation. This discrepancy between theory and practice underscores the challenges of applying Top-k loss strategies directly within these constrained domains.

Further examining convergence, ref. [15] introduced an averaged Top-k loss formulation and proved that it maintains convexity, provided that all individual loss functions are convex. Under these conditions, the optimization problem admits a global minimum. This theoretical result also supports our formulation: if each pixel-wise or instance-wise loss function is convex, then the proposed sigmoid-based selection mechanism—denoted as $\zeta_{\alpha }\left(\cdot \right)$ in Equation (7)—produces non-zero weighting factors that preserve convexity in the final weighted sum. Consequently, the entire parametric form remains convex, and standard convergence guarantees apply.

However, similar to the earlier theoretical limitations, this conclusion also hinges on assumptions that may not fully hold in practice. Specifically, when the aggregation is performed over a small number of terms—as in Top-k strategies with low *k*—any deviation from convexity in the constituent terms becomes more impactful. In such cases, the convergence behavior may diverge from the theoretical expectation, making training less stable or more sensitive to initialization and hyperparameter choices.

An additional consideration relates to the core motivation behind using Top-k ranking-based losses. The central idea is that the hardest examples—the ones associated with the highest loss values—are the most informative for learning, as they impose a stronger learning signal than the average case. Intuitively, such hard examples behave more like outliers, residing further from the bulk of the data distribution. This deviation makes their loss values less predictable and potentially more irregular. As a result, focusing learning solely on these difficult cases raises questions about the smoothness and consistency of the optimization landscape, especially in the absence of regularization or smoothing techniques.

Despite this concern, our empirical observations suggest that convergence can still be reliably achieved, particularly when *k* is moderately large and a smoothing mechanism is incorporated. The smoothing reduces gradient noise and stabilizes the learning dynamics, even when the loss is concentrated on a subset of harder examples.

In contrast, the “bottom all but σ” strategy, which excludes only a small portion of the loss values while retaining the majority, has a less disruptive impact on convergence. By discarding only a minor subset of pixel-wise losses (typically those associated with annotation errors or noise), the optimization becomes more robust, and convergence is often facilitated. In essence, by filtering out the least trustworthy data points, this strategy simplifies the learning process rather than complicating it.

## 4. Implementation and Databases

### 4.1. Implementation Aspects

The code has been implemented in PyTorch and is publicly available at https://zenodo.org/records/15017434 (accessed on 13 March 2025). All models were trained on a single NVIDIA RTX A4000 GPU with 16 GB of memory. The learning algorithm was Adam. All backbone encoder models have been pretrained on ImageNet. The batch size depends on the architecture model used and was up to 16.

We tested the proposed paradigm in three different applications. The first one was segmenting the burned skin area on the BAMSI database [32]. The second problem was the segmentation of fetal abdominal structures in ultrasound images. These first two exhibit significant noise and represent difficult problems. Thirdly, to evaluate more thoroughly the proposed method, we have considered the public medical image segmentation database of Automated cardiac diagnosis challenge (ACDC); it contains MRI scans and, compared to the first two databases, the segmented objects are more clearly distinguishable.

When experimenting with convolutional networks (UNet3+ [33] and DeepLab-v3 [34]), we have used a learning rate of $10^{-5}$. For the tests with visual transformers, the code is developed from the work of [35], the model encoders are PVTv2-b2 [36] and Small CascadedMERIT [35], and the learning rate was $10^{-4}$.

### 4.2. Databases

**Figure 5:**
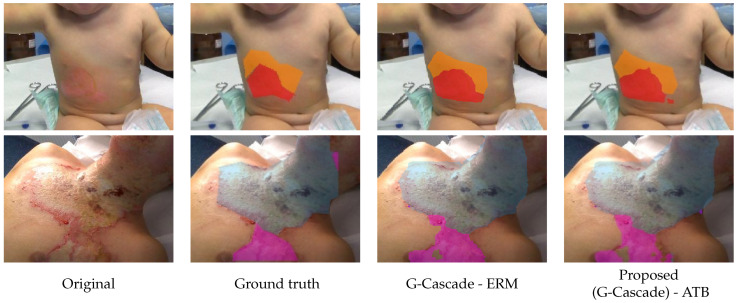
Example of segmentation of burn skin area on the BAMSI database. Each row represents a case; columns represent the original color image, the ground truth segmentation, the result obtained by the G-Cascade—ERM from [35] and the proposed approach. Annotations and predictions for the burn grades are color-coded: grade 1 with orange, grade 2a with red, grade 3 with magenta and grade 4 with cyan.

**Figure 6:**
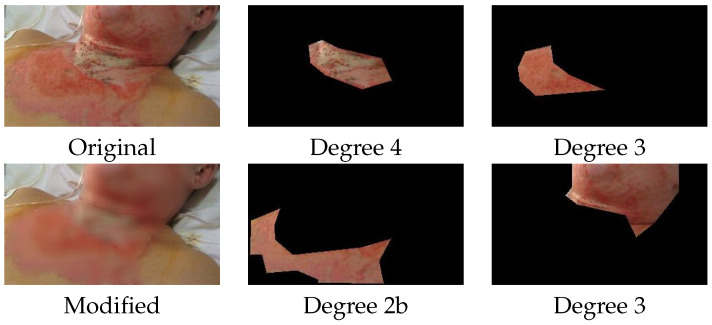
Synthetic homogenization of burned areas. We have artificially smoothed the burned areas to make limits clearer and especially to increase homogeneity inside the annotated areas. In this way, we have minimized the annotation error at pixel level. The burned areas from the right hand side are in the original form.

**Burn Assessment by MultiSpectral Imaging—BAMSI**. The database was assembled over numerous months in hospital conditions in the burns and plastic surgery unit of a pediatric clinic. The database contains color images acquired by medical personnel who lacked advanced training in acquiring digital images. Trained medical staff selected and annotated the image sections deemed essential for diagnosis. Compared to the initial version of the database [32], where the considered problem was image classification (i.e., using only the burn crop and one label with the burn degree), now we use full image masks. We also dropped the infrared images as they offer poor resolution. Original image resolution is 1664×1248 pixels, yet the annotation and working resolution is 320×240 pixels. The dataset includes 611 images, depicting burn injuries of varying severity from 55 pediatric patients. The patients’ ages were from 8 months to 17 years, with an average age of 4 years, a median of 2 years, and a standard deviation of 4 years. The timing of image capture ranged from one day to sixty-two days post-incident, mainly occurring within the first ten days post-burn. Permission from all patient parents or guardians has been granted to use the anonymized images for research purposes only. Examples of images from this database are presented in Figure 1, Figure 5 and Figure 6.

Burn-specialized (i.e., plastic) surgeons manually labeled each color image crop, identifying areas correlating with burn wounds and their severity. An image might exhibit multiple annotation masks, each labeled with different burn severity levels, resulting in 1634 delineated areas of interest (annotation masks) that range from the lightest burns to 4th degree burns. Additionally, areas of healed wounds were also marked. The classes thus, are: background, healed, 1st, 2nd-a, 2nd-b, 3rd, 4th.

**Figure 7:**
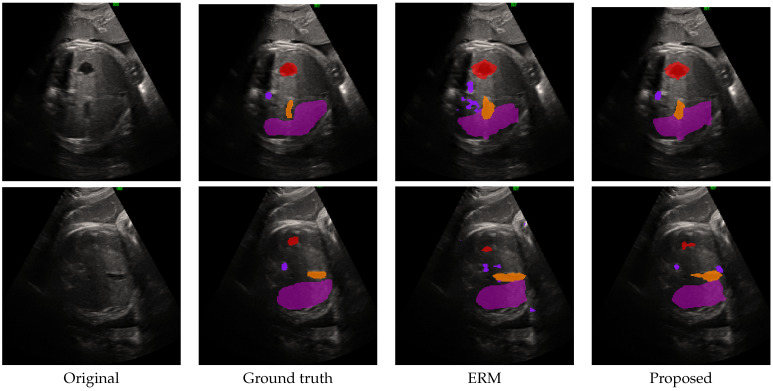
Example of segmentation of fetal abdominal structures in ultrasound imaging. Each row represents a case. With respect to annotations, with magenta is the “stomach”, orange is the “liver”, red is the “artery”, while with violet is the “vein”.

**Fetal Abdominal Structures in Ultrasound images**—FASU [37] consists of 1588 images from 169 subjects available at https://data.mendeley.com/datasets/4gcpm9dsc3/1 (accessed at 15 October 2024). The subjects were pregnant women aged 18 years or older, either in labor or scheduled for delivery at the Maternity of University Hospital Polydoro Ernani de São Thiago in Florianopolis, Santa Catarina, Brazil. This encompassed patients scheduled for labor induction, cesarean section, and those facing pregnancy complications. Clinicians used a standardized acquisition protocol to obtain ultrasound images, capturing a common axial section of the fetal abdomen circumference. Measurements were taken at the widest part of the fetal abdomen, spanning the liver. In this section two expert clinicians marked key components, like the abdominal aorta artery, intrahepatic umbilical vein, the stomach, and the liver area. Original images (exhibiting the typical arc shape) have 1024×768 pixels. The train/test set was chosen taking into account the subject: 75% (≈1300 images) for training and the rest for testing. All images from one subject are either in train, or in test. Examples of images from this database may be seen in Figure 7.

**Figure 8:**
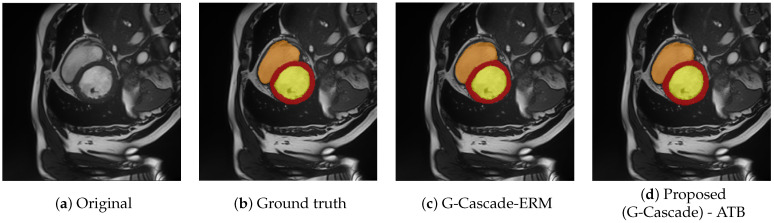
Examples of images from ACDC challenge. The original image and respectively the legend of annotations (**a**) ground truth annotations (**b**), results from G-Cascade [35], that uses an ERM aggregation (**c**) and the result with the proposed aggregation (**d**). Note the objects that are sought have clearer boundaries than in the case of burns.

**Automated cardiac diagnosis challenge**—ACDC consists of MRI scans from various patients. The MRI images are captured during a breath hold, with multiple short-axis slices documenting the heart from the base to the apex of the left ventricle, featuring a slice thickness ranging from 5 to 8 mm. The spatial resolution of these short-axis in-plane images varies between 0.83 and 1.75 mm^2^ per pixel. Each patient’s scan is manually labeled to identify the left ventricle (LV), right ventricle (RV), and myocardium (MYO). The dataset organization follows [7,35] using a division into 70 training cases (1930 axial slices), 10 validation cases, and 20 testing cases and reporting the average Dice Score (DSC). Example of images from this database may be seen in Figure 8. For our method, the training images, in the sense of Equations (8) and (9) are the slices, and I=1930.

## 5. Results

The evaluation considers, sequentially, each database and the associated problem. Burn area segmentation and ultrasound fetal abdominal structures are deemed harder problems due to the increased noise within the images and the poorer separation of objects of interest. The problem based on MRI scans is somehow easier and are covered by a significant number of previous works; here the top performance has been clearly established by the use of transformer based models, so we will focus only on the transformer approaches. For each problem, we also perform experiments showing the impact of various parameters and strategies of the proposed methodology.

### 5.1. Strategies—Ablation

Fundamentally, the proposed framework assumes selecting the top harder images and ignoring the most difficult pixels in those images. The selection is based on several parameters. With respect to the various parameters required for the top best and respectively the bottom worst, there are different choices that may be set during the iterative learning process. Intuitively, one has to start by doing the forward step on all data instances, in order to have computed all the losses, before having the possibility to rank these losses. Furthermore, smoothing is necessary in order to prevent oscillation (i.e., non convergence). The used choices are:

- With respect to the Top-k images:
  - Using all images. This is the standard Empirical Risk Minimization and will be denoted by “ERM”.
  - Setting $i_{0}=N-k$, with k=10%N—fixed from epoch 2 onwards. This is the standard average Top-k loss and will be denoted by “${\mathrm{AT}}_{k}$”.
  - Starting with k=N, $i_{0}=0$ and decreasing gradually until k=5%N, $i_{0}=95\%N$. This will be denoted by “${\mathrm{AT}↓}_{k}$”.
- With respect to the indicator function approximation, which is used and needed only at image level:
  - No approximation—using the indicator function directly. This corresponds to α=+∞.
  - Fixed approximation—α is fixed at a relatively large value α=20. This is denoted by “Sm” (constant smoothing).
  - Variable approximation—α decreases over iterations, such that it may compensate for sharp Top-k. Start at α=20 and decrease to α=1. This is denoted by “Sm↑” (smoothing increases).
- With respect to the pixels, where the preferred strategy is to use “Bottom all but σ”:
  - All pixels are used—the classical approach.
  - Pixels within top-k=10% are the only ones used. This is the Average Top-k at pixel level and is denoted by “$T_{k}$”.
  - Bottom “all but σ” are used. This is the proposed approach and will be denoted by “$B_{\sigma }$”.

For particular strategies, the effect is that of removing a certain selection from the loss aggregation, either at pixel level, or at image level. Thus ablation is performed. The tests with various strategies have been implemented for all experiments, although more testing is strictly related to the segmentation of burned skin.

### 5.2. Skin Burns Segmentation on BAMSI Dataset

Visual results may be seen in Figure 5. On this database, the convolutional architecture UNet3+ [33] and respectively DeepLab-V3 [34,38] are used as baselines. Furthermore, we report results with the architectures used by [26] and, respectively, by [28] for the same problem of burn skin segmentation. In addition we have tested the powerful solution from [35]. Parameters from Equation (12) are $\lambda_{CE}=0.3$ and $\lambda_{DC}=0.7$.

Results may be seen in Table 1. The improvement with the proposed method is significant over the ERM-baselines.

#### Parameter and Choice Importance

In this context, we have investigate multiple aspects of the proposed method. Various strategies for aggregation at both the image and pixel levels are presented in Table 1. For cases when we used only the top *k* pixels (denoted by $T_{k}$) the method focuses only on noisy annotated pixels which hurts the convergence.

It is important to note that derivative smoothing by approximation is necessary for convergence. Additionally, increasing approximation proves beneficial when used alongside the reduction of the number of images. This can be seen as a reinterpretation of the curriculum learning strategy. The use of only images with Top-k losses is too abrupt and confuses the learning process.

Another point of emphasis is that the proposed “bottom all but σ” strategy leads to noticeable increase in performance, as compared to the case when all the pixels are used. We will discuss this briefly in the next paragraph.

**Importance of “bottom all but **σ**”. Homogenization of the burned area.** We hypothesized that the “all but σ” choice is beneficial if pixel labels are noisy. We cannot override the expert and correct the annotation, but we can alter the data to match the labels. To do this, we have replaced all the pixels inside the annotated masks with a very blurred version of them. An example image may be seen in Figure 6. This process is in fact a synthetic homogenization of the burned area, but using specifically the annotated mask as boundaries. Images have been modified into this manner for both training and testing. The backbone architecture was UNet3+ and the performance of various alternatives are shown in Table 2. In this case, the ”bottom all but σ” strategy is no longer beneficial as it hurts the performance. We consider this a consequence of the fact that all the pixels are labeled “correctly”.

### 5.3. Fetal Abdominal Structures Segmentation on FASU Database

Visual examples of the original ultrasound images, ground truth and predictions may be seen in Figure 7. As one can see, the problem is harder and the results are less accurate than in other problems. This observation is confirmed by the objective evaluation presented in Table 3. Again, we report comparison for multiple architectures. To optimize the performance, uneven weights were needed: 2 for the larger “liver” and “stomach” and 4 for the smaller and more rare “artery” and “vein”. Parameters from Equation (12) are $\lambda_{\mathrm{CE}}=0.15$ and $\lambda_{\mathrm{DC}}=0.85$.

The observation that this problem is harder is confirmed both visually, where one needs significant experience to locate the fetal structures, and objectively. To evaluate the performance we have tested with four baseline architectures, out of which three are based on convolutional layers (UNet, UNet3+, DeepLab-v3) while the fourth (G-Cascade with PVT encoder) uses the more recent transformer paradigm. The baselines have been achieved using the ERM aggregation. Following the baseline, the same architectures were used, but combined with various strategies based on the proposed “Top-k, All but σ” paradigm. Based on the results from the burned skin, we focused on cases which were expected to converge. Reported results showed that in all the tested cases, this strategy showed to be beneficial as it improve with respect to its baseline.

The best performance, according to three metrics, is obtained by using the Top-k images with smoothing and all but σ pixels. The accuracy, Dice score and mIoU noted significant increases. The only metric which has not noted any improvement was the Hausdorff distance threshold, which was due to a poorer centering in the latter case.

### 5.4. Evaluation on MRI Scans

To better evaluate the performance of the proposed method, we have considered testing on the public database of ACDC challenge which is based on MRI scans. The problem is easier and witnessed more proposed solutions than the previously approached cases. The baseline methods on these databases are PVT and MERIT G-Cascades visual transformer models proposed by [35]. Parameters from Equation (12) are $\lambda_{\mathrm{CE}}=0.3$ and $\lambda_{\mathrm{DC}}=0.7$. Results may be followed in Table 4 where it can be noticed that the proposed envelope for the loss function aggregation always brings improvements.

Overall, the segmentation performance on these datasets is noticeably higher, reflecting the relatively lower complexity of the underlying tasks. This observation is consistent with intuitive expectations: a visual inspection of Figure 1 and Figure 8 reveals clearer separation between the different anatomical classes and reduced intra-class variability. In particular, in the ACDC dataset (Figure 8), the boundaries of the target structures are well defined and easily distinguishable—even to a non-expert observer—making the segmentation task more straightforward.

Although the application of the proposed envelope over the loss function does lead to improved results in these simpler cases, the magnitude of improvement is less pronounced compared to its effect on more complex tasks, such as segmentation of burned skin regions or fetal abdominal structures in ultrasound images. In those more challenging scenarios, the benefits of the method are more substantial due to greater variability, ambiguous boundaries, and noisy pixel-level annotations.

The key factor driving the observed improvements across tasks is the model’s ability to focus on more difficult examples during training, a feature made effective through the combined use of top-k and smoothing strategies. Among these, the smoothing component plays a particularly crucial role: without it, the performance consistently deteriorates, highlighting its necessity for stable optimization.

In contrast to the noisy labels often encountered in ultrasound or burned skin datasets, MRI scans—such as those in the ACDC collection—provide cleaner, more accurate pixel-level annotations. As a result, the “all but σ” pixel-level loss filtering strategy has a more neutral impact in this context, neither significantly helping nor harming performance.

## 6. Discussion

In this paper we proposed the framework for a loss function to be used in segmentation problems, which is based on ranking and selecting values according to prior information about the problem. The assumption was that object classes are correctly identified, but the boundaries of the objects are inexact; this translated into a high accuracy at image level annotations and noise at pixel level annotation. We developed and evaluated the proposed framework first for burned skin image segmentation, where we showed greater increase over standard (ERM) approaches. We regard the burned area segmentation as a difficult problem because even specialists have a rather modest upper limit for the annotation performance and the overall performance is much lower than on other benchmarks. Furthermore, it is easy to confuse the correct classes of different regions based only on the local information and real-life observers aggregate global information, such as body part recognition, to have a better understanding of the area. Next, we continued the evaluation on another difficult problem, that is based on ultrasound imaging and refers to the segmentation of abdominal structures in unborn fetuses. While the solution is imperfect, the proposed strategy noticeably helped, no matter if the baseline architecture was based on convolutional networks or visual transformers.

To further investigate the capabilities of the method, we evaluated it on another established benchmark comprising MRI scans in conjunction with visual transformer models. In this problem, the separation between the different objects is easier, both visually and based on top performance, and we consider this problem as being simpler. Even in this case, the proposed framework managed to improve the overall performance with a noticeable margin, arguing for the strength of the method.

The here-proposed method may be combined with preprocessing techniques such as the one proposed by Subramani et al. [41], which improves the visibility of clinically relevant structures and can make Top-k selection more meaningful and less susceptible to noise. However, such preprocessing introduces additional computational cost, may create artifacts, and carries the risk of distribution shift if not applied consistently at inference time. When combined carefully, the two strategies have the potential to yield more accurate and robust segmentation results, but require careful tuning of k, σ, and enhancement parameters to avoid overfitting to artifacts or noisy labels. Also the Top-k Bottom-all-but-σ approach may complement segmentation pipelines that rely on handcrafted feature extraction. For example, pixel selection within a U-Net or CNN-based segmentation stage could be replaced by this method, enabling approaches like that of Ragupathy et al. [42] or the FGChO-based ShCNN [43] to refine their focus on diagnostically important regions while avoiding the influence of noisy pixels. Nonetheless, integration would require reconciling differences in task granularity (as in ref. [42]) and managing additional computational overhead (as in [43]).

Also we would like to point that it was current choice to construct our solution in conjunction with Cross Entropy and Continuous Dice Loss; other alternative may be followed. Also, the ordering (ranking) principle applied here is derived directly but alternatives do exist. Focal loss [20] inherently is a re-weighting of images and the functionality can be integrated with principle proposed in our paper which is to concentrate on on hardest images (Top-k) and–less noisy pixels (Bottom all but σ). Pixel ranking behavior may be derived from Unified Focal Loss [44].

The method is primarily designed to handle noisy pixel-level annotations while assuming that image-level labels are reliable. This is because the Top-k Bottom-all-but-σ approach leverages image-level labels to guide pixel selection and loss sorting, focusing training on the hardest—but still correctly labeled—pixels. If image-level labels themselves are noisy or imperfect, this foundational assumption is violated, which can degrade performance since the model may prioritize misleading pixel regions or incorrect samples overall. If both pixel- and image-level annotations are noisy, combining our Top-k Bottom-all-but-σ pixel selection with methods that estimate or correct image-level label noise could offer a robust framework for improving segmentation performance under real-world annotation imperfections.

### Limitations

We have demonstrated that the proposed method can significantly improve tasks with poor pixel-level separation, largely due to the strategy of ignoring hard pixels. Additionally, there is a marginal improvement in tasks with accurate annotations, particularly because of the strategy of selecting the “worst” image instances in the later stages of learning. In machine learning, the “no free lunch” theorem [45] points to the fact that there is no algorithm that is optimal for all problem topologies, since prior assumptions will make the difference between degrees of performance. This proposal is no exception; if all data, both at pixel level and at image level, are equally important and accurately annotated, there is no reason to assume that ranking data helps.

However, we argue that in the general medical image segmentation problem, the envisaged scenario is very likely: databases are small, although on an increasing trend, and contain both easy examples (used in medical teaching to explain the basics) and hard ones, which are needed in the later stages of learning (both for machines and medical students) and annotations at pixel level are sometimes noisy, the root cause being the limited time that experts allocate for labeling. The proposed method may act as a guideline, too: if it increases the segmentation performance, especially due to ignoring some of the pixel labels, than it might be a good idea for experts to revisit pixel-level annotation and curate them.

## 7. Conclusions

The proposed envelope applied over the loss function introduces a novel mechanism that enhances model training by selectively emphasizing certain loss values. Specifically, it incorporates two complementary strategies: at the image level, it identifies and focuses on the top-k highest loss values, effectively prioritizing the most challenging regions of the image; at the pixel level, it implements a filtering approach that disregards all but a small proportion, denoted by σ, of the largest individual pixel-wise losses. This dual-selection mechanism is further augmented with a smoothing strategy to ensure training stability and robustness.

Together, these components contribute significantly to performance improvements across a variety of medical image segmentation tasks. The benefits of this approach are especially pronounced in more challenging scenarios, where the segmentation problem is inherently difficult or where the quality of pixel-level annotations is degraded by noise or inconsistencies. In such cases, the method’s ability to concentrate learning on the most informative and reliable features proves particularly advantageous.

Importantly, the effectiveness of the proposed method has been thoroughly validated using both convolutional neural networks (CNNs) and vision transformer (ViT) architectures, demonstrating its flexibility and broad applicability across different deep learning frameworks for medical imaging.

## Figures and Tables

**Table 1:** Results of Burn area (BAMSI) segmentation. The best results are shown in bold. “nc” stands for not converged. In the top part are listed the results using aggregation according to the ERM principle. In the bottom part the results obtained according to various ranking strategies are listed. CE-stands for cross entropy, while Dice stands for the Dice score. The acronyms of different variants are explained in the “Strategies” subsection.

Architectures/Loss	Accuracy ↑	Dice ↑	mIoU ↑
MaskRCNN-CE [26]	72.38	35.90	28.16
UNet-CE [28]	74.26	36.01	28.85
UNet3+-CE	84.14	44.69	37.76
UNet3+-CE+Dice	85.45	45.85	38.15
DeepLab-v3-CE	86.37	43.15	39.39
DeepLab-v3-CE+Dice	86.72	43.58	40.05
G-Cascade-CE	76.81	39.72	33.81
G-Cascade-CE+Dice [35]	89.71	66.72	48.14
UNet3+-CE+Dice+${\mathrm{AT}}_{k}$	nc	nc	nc
UNet3+-CE+Dice+${\mathrm{AT}↓}_{k}$	81.43	39.81	35.24
UNet3+-CE+Dice+${\mathrm{AT}↓}_{k}$+Sm	81.57	39.89	37.64
UNet3+-CE+Dice+${\mathrm{AT}↓}_{k}$+Sm↑	84.12	45.24	39.85
UNet3+-CE+Dice+${\mathrm{AT}↓}_{k}$+Sm↑+$T_{k}$	nc	nc	nc
UNet3+-CE+Dice+${\mathrm{AT}↓}_{k}$+Sm↑+$B_{\sigma }$	89.27	48.88	43.81
DeepLab-v3-CE+Dice+${\mathrm{AT}}_{k}$	nc	nc	nc
DeepLab-v3-CE+Dice+${\mathrm{AT}↓}_{k}$	83.21	40.93	38.88
DeepLab-v3-CE+Dice+${\mathrm{AT}↓}_{k}$+Sm	90.27	45.17	43.16
DeepLab-v3-CE+Dice+${\mathrm{AT}↓}_{k}$+Sm↑+$T_{k}$	nc	nc	nc
DeepLab-v3-CE+Dice+${\mathrm{AT}↓}_{k}$+Sm↑+$B_{\sigma }$	92.26	46.18	44.18
G-Cascade-CE+Dice+${\mathrm{AT}↓}_{k}$+Sm	92.14	67.17	49.02
G-Cascade-CE+Dice+${\mathrm{AT}↓}_{k}$+Sm↑+$B_{\sigma }$	**94.26**	**69.23**	**51.09**

**Table 2:** Results on the BAMSI database with synthetic and homogeneous burns.

Architectures/Loss	Accuracy ↑	Dice ↑	mIoU ↑
UNet3+-CE+Dice	96.81	72.85	64.15
UNet3+-CE+Dice+${\mathrm{AT}↓}_{k}$	87.01	61.81	59.24
UNet3+-CE+Dice+${\mathrm{AT}↓}_{k}$+Sm	94.57	73.89	67.64
UNet3+-CE+Dice+${\mathrm{AT}↓}_{k}$+Sm↑	97.16	73.25	64.64
UNet3+-CE+Dice+${\mathrm{AT}↓}_{k}$+Sm↑+$T_{k}$	nc	nc	nc
UNet3+-CE+Dice+${\mathrm{AT}↓}_{k}$+Sm↑+$B_{\sigma }$	93.12	69.88	55.81

**Table 3:** Fetal abdominal structures segmentation on FASU ultrasound database. The best results are shown in bold. In the top part are listed the results using aggregation according to the ERM principle, while in the bottom part are listed the results obtained according to various ranking strategies. CE-stands for cross entropy, while Dice stands for the Dice score. Again, the acronyms of different variants are explained in the “Strategies” subsection.

Architectures/Loss	Accuracy ↑	Dice ↑	Average HD95 ↓	mIoU ↑
UNet-CE+Dice [39]	29.46	15.26	30.43	10.81
UNet3+-CE+Dice [33]	31.45	16.58	29.15	14.61
DeepLab-v3-CE+Dice [34]	36.72	23.85	29.08	24.34
G-Cascade-CE+Dice [35]	65.33	55.03	**24.97**	46.63
UNet3+-CE+Dice+${\mathrm{AT}↓}_{k}$+Sm↑	32.18	18.41	29.37	16.11
UNet3+-CE+Dice+${\mathrm{AT}↓}_{k}$+Sm↑+$B_{\sigma }$	33.15	19.51	29.21	17.89
DeepLab-v3-CE+Dice+${\mathrm{AT}↓}_{k}$+Sm	36.18	23.47	29.18	25.87
DeepLab-v3-CE+Dice+${\mathrm{AT}↓}_{k}$+Sm↑+$B_{\sigma }$	38.12	26.17	28.88	28.17
G-Cascade-CE+Dice+${\mathrm{AT}↓}_{k}$+Sm	66.88	56.41	25.16	48.02
G-Cascade-CE+Dice+${\mathrm{AT}↓}_{k}$+Sm↑+$B_{\sigma }$	**67.44**	**57.01**	25.07	**48.88**

**Table 4:** Results of ACDC segmentation. The best results are shown in bold. The evaluation is overall (marked with DICE) and specifically for left ventricle (LV), right ventricle (RV), and myocardium (MYO).

Architectures/Loss	DICE	RV	MYO	LV
R50+UNet [7]	87.55	87.10	80.63	94.92
R50+AttnUNet [7]	86.75	87.58	79.20	93.47
ViT+CUP [7]	81.45	81.46	70.71	92.18
R50+ViT+CUP [7]	87.57	86.07	81.88	94.75
TransUNet [7]	89.71	86.67	87.27	95.18
MISSFormer [40]	90.86	89.55	88.04	94.99
Cascaded MERIT [35]	91.85	90.23	89.53	95.80
PVT-GCASCADE [35]	91.95	90.31	89.63	95.91
MERIT-GCASCADE [35]	92.23	90.64	89.96	96.08
PVT-GCASCADE +${\mathrm{AT}}_{k}$	90.82	88.43	89.15	94.89
PVT-GCASCADE +${\mathrm{AT}↓}_{k}$+Sm	91.96	90.54	89.53	95.82
PVT-GCASCADE +${\mathrm{AT}↓}_{k}$+Sm↑+$T_{k}$	92.07	90.17	90.16	95.87
PVT-GCASCADE +${\mathrm{AT}↓}_{k}$+Sm↑+$B_{\sigma }$	92.11	90.68	89.93	**96.32**
MERIT-GCASCADE +${\mathrm{AT}}_{k}$	91.55	89.55	89.01	96.08
MERIT-GCASCADE +${\mathrm{AT}↓}_{k}$+Sm	91.99	89.64	90.46	95.88
MERIT-GCASCADE +${\mathrm{AT}↓}_{k}$+Sm↑+$T_{k}$	92.18	89.92	**90.51**	96.12
MERIT-GCASCADE +${\mathrm{AT}↓}_{k}$+Sm↑+$B_{\sigma }$	**92.37**	**90.75**	90.12	96.24

## Data Availability

Data Availability Statement: The following datasets were used in this study: (1) BAMSI (Burn Assessment by MultiSpectral Imaging) Database which is not readily available because of the patients’ confidential agreement; (2) FASU (Fetal Abdominal Structures in Ultrasound images) Database openly available at https://data.mendeley.com/datasets/4gcpm9dsc3/1, accessed on 17 August 2025 (3) ACDC (Automated cardiac diagnosis challenge) Database openly available at https://www.creatis.insa-lyon.fr/Challenge/acdc/databases.html, accessed on 17 August 2025.

## Abbreviations

The following abbreviations are used in this manuscript:

ATK	Average Top k
ATB	Average Top Bottom
BAMSI	Burn Assessment by MultiSpectral Imaging
BN	Batch Normalization
CE	Cross Entropy
CNN	Convolutional Neural Network
ERM	Empirical Risk Minimization
FASU	Fetal Abdominal Structures in Ultrasound
MRI	Magnetic Resonance Imagining
PVT	Pyramid Visual Transformer
ViT	Visual Transformer
